# Effects of agri-environmental schemes on farmland birds: do food availability measurements improve patterns obtained from simple habitat models?

**DOI:** 10.1002/ece3.1125

**Published:** 2014-06-11

**Authors:** Carlos Ponce, Carolina Bravo, Juan Carlos Alonso

**Affiliations:** Department of Evolutionary Ecology, Museo Nacional de Ciencias Naturales, CSICJosé Gutiérrez Abascal 2, Madrid, E-28006, Spain

**Keywords:** Agri-environmental scheme, agricultural intensification, biomass, habitat management, steppe birds, wildlife conservation

## Abstract

Studies evaluating agri-environmental schemes (AES) usually focus on responses of single species or functional groups. Analyses are generally based on simple habitat measurements but ignore food availability and other important factors. This can limit our understanding of the ultimate causes determining the reactions of birds to AES. We investigated these issues in detail and throughout the main seasons of a bird's annual cycle (mating, postfledging and wintering) in a dry cereal farmland in a Special Protection Area for farmland birds in central Spain. First, we modeled four bird response parameters (abundance, species richness, diversity and “Species of European Conservation Concern” [SPEC]-score), using detailed food availability and vegetation structure measurements (*food models*). Second, we fitted new models, built using only substrate composition variables (*habitat models*). Whereas habitat models revealed that both, fields included and not included in the AES benefited birds, food models went a step further and included seed and arthropod biomass as important predictors, respectively, in winter and during the postfledging season. The validation process showed that food models were on average 13% better (up to 20% in some variables) in predicting bird responses. However, the cost of obtaining data for food models was five times higher than for habitat models. This novel approach highlighted the importance of food availability-related causal processes involved in bird responses to AES, which remained undetected when using conventional substrate composition assessment models. Despite their higher costs, measurements of food availability add important details to interpret the reactions of the bird community to AES interventions and thus facilitate evaluating the real efficiency of AES programs.

## Introduction

The demand of more food and biofuel (Tilman et al. 2011; Miyake et al. 2012) from modern agricultural activities has caused the decline of many species inhabiting farmland areas (Donald et al. 2001). Increased use of chemicals (pesticides, fertilizers, etc.), loss of noncropped habitats and loss of crop diversity are some of the most important factors affecting plant and animal populations in these ecosystems (Chamberlain et al. 2000; Vickery et al. 2001; Robinson and Sutherland 2002; Benton et al. 2003). However, agri-environmental schemes (AES, hereafter) are intended to reverse the environmental impacts of modern farming techniques on biodiversity (Stoate et al. 2009). It is generally accepted that an increase in habitat heterogeneity has a positive influence on biodiversity (Wuczyński et al. 2011). The European Union and the United States of America have spent several billion dollars in AES programs (Kleijn et al. 2006; Gabriel et al. 2010), but their effectiveness is still somehow questioned because different studies have reported contradictory results (Tscharntke et al. 2005; Kleijn et al. 2006). These differences may have been due to differences in the scale of study, with most clearly positive effects at local scales (Perkins et al. 2011) compared with larger scales (Verhulst et al. 2007; Davey et al. 2010), or in studies designed to enhance certain declining species (Wilson et al. 2009; Kleijn et al. 2011). AES are usually implemented at field scale, without controlling for the spatial complexity (vegetation structure and substrate diversity around managed fields) that affects the variables under study (Kleijn and Sutherland 2003; Tscharntke et al. 2005; Concepción et al. 2008; Gabriel et al. 2010; Winqvist et al. 2011). AES design and the research on their effectiveness usually focus on responses of just one or a few species (Breeuwer et al. 2009), although other species (MacDonald et al. 2012) or functional groups (granivorous, insectivorous, etc.; Henderson et al. 2000) may also obtain benefits. AES studies rarely analyse the responses of the whole bird community, ignoring that biodiversity maintenance should be a priority (Ekroos et al. 2014). Finally, although habitat and feeding requirements of species change through the year (Marfil-Daza et al. 2013), most AES studies evaluate effectiveness during a single season.

In the present study, we investigated the effects of an AES on a steppe bird community in a dry cereal farmland area in central Spain. We did this by analyzing various abundance and diversity parameters, which define direct bird responses of the farmland bird community to the AES (Díaz et al. 2006). The populations of 17 dry farmland bird species present in Spain are rapidly declining (Escandell et al. 2011), even faster here than in other European countries (EBCC 2010). Despite this, Spain still holds the most important breeding populations of several species classified as endangered at a continentwide scale, for example, the pin-tailed sandgrouse (*Pterocles alchata*), lesser kestrel (*Falco naumanni*) or great bustard (*Otis tarda*). Also, Spain holds significant wintering populations of common European species like the meadow pipit (*Anthus pratensis*) or the skylark (*Alauda arvensis*). Thus, Spain has the highest impact on the European Farmland Bird Index (EFBI), an indicator for biodiversity health on farmland areas (Butler et al. 2010a,b2010b). As the scheme prescriptions and measures of our AES (Table 1) were designed for a broad spectrum of bird species, the results of this study may be considered as widely applicable for managers and ecologists in general.

**Table 1:** List of field types and prescriptions of the agri-environmental scheme (AES) implemented in the study area

Field type	Short name	Origin	Scheme prescriptions
Legume	LegAES	AES	Growing organic legumes. Sowing seeds on ploughed fields (190 kg/ha) in October with a mixture of up to 20% cereal seed. No use of dressed seed. No agricultural activities (weed and arthropod control, tillage tasks, fertilizer applications) from sowing to harvest after 10 July^1^
	LegNAT	Natural, non-AES farming	Not applicable
Cereal stubble	CerStubbleAES	AES	Maintenance of cereal stubble. No agricultural activities from usual harvest date from June–July to 31 December and from 1 April to 1 July. Tillage tasks allowed from 1 January to 31 March without use of herbicides or insecticides.
	CerStubbleNAT	Natural, non-AES farming	Not applicable
Fallow	FallowAES	AES	Interruption of the cereal production for ≥1 years. No agricultural activities (weed and arthropod control, tillage tasks, fertilizer applications) are allowed from July to the next July. The agreement can be renewed annually. Fields included in this AES must have been used for agricultural purposes on the last three years
	FallowNAT	Natural, non-AES farming	Not applicable
Legume stubble	LegStubbleAES	AES	No scheme prescriptions. It comes from the LegAES measure
	LegStubbleNAT	Natural, non-AES farming	Not applicable
Cereal crop	CerNAT	Natural, non-AES farming	Not applicable
Plough	Plough	Natural, non-AES farming	Not applicable
Plough with sprouted weeds	Plough2	Natural, non-AES farming	Not applicable
Edge	Edge	Natural, non-AES farming	Not applicable

1 Although AES limit the harvest date from 10 July, we accepted farmers harvesting legume fields earlier (but always after 31 June) to feed sheep or to collect the seed for the following sowing season.

The AES was funded by a program of preventive, corrective, and compensatory measures to balance the impact of the M-50 and R-2 highways on the population of great bustards and other steppe-land birds in the Important Bird Area (IBA) Talamanca- Camarma and the Site of Community Importance cuenca de los ríos Jarama y Henares. The two highways were built in the inner border of the Special Protection Area (SPA) 139 Estepas cerealistas de los ríos Jarama y Henares, which is included in the IBA. To implement the AES, first we contacted with agricultural agents to prepare a meeting with farmers from the SPA. Second, we evaluated all fields farmers offered to be included in the AES following suitability criteria such as distance to power lines, fences, towns, etc. Third, once a field was accepted and AES measure implemented on it, we made periodic checks of each field. Finally, payments to farmers were performed by the company operating the highways.

However, our main purpose was not just to test the AES effectiveness. Our first major objective was to explore the effects of a differing quality of the field data commonly used to investigate bird responses to AES. We did this by comparing the predictive capacity of response models based on simple habitat measurements (called *habitat models* hereafter) with that of models also based on habitat variables and much more detailed food availability and vegetation structure measurements (*food models* hereafter). We also analyzed the cost in terms of money and effort spent on each set of models, because it is expected that the amount of time and funds invested should correspond to the quality of the results obtained. Most AES effectiveness studies have been carried out with relatively low investment in field work, often using the composition of substrates selected by the birds before and after AES implementation (see review in Kleijn and Sutherland 2003). The only conclusion that can be drawn from these studies is the positive, neutral or negative effect of the AES on individual behavior, whereas in most cases the ultimate causes, processes and population level consequences remain largely unknown. However, it has been suggested that the association between bird species and their habitat is determined by the quantity and quality of the resources provided (functional space available to a species), not only the habitat per se (Boyce and McDonald 1999; Butler and Norris 2013). For example, it is currently admitted that agriculture intensification has determined massive declines of farmland bird species, and these declines are due to different processes as reduced food resources (food availability hypothesis, Newton 2004), reduced refuge quality (refuge and nesting hypothesis; Benton et al. 2003), or both in some cases (Campbell et al. 1997; Butler et al. 2007, 2010a,b2010b). Some studies on these questions are species-specific and do not consider the whole bird community (Breeuwer et al. 2009; Bretagnolle et al. 2011). However, it is considered that biodiversity loss can compromise many ecosystem services (Cardinale et al. 2012) and the impacts of species loss on primary productivity are comparable with impacts from climate warming (Hooper et al. 2012; Tilman et al. 2012).

Our second main objective was to compare different bird responses: abundance, richness, diversity and SPEC-score, an index based on the Species of European Conservation Concern (BirdLife International 2004) among three periods of the annual cycle: wintering, mating, and postfledging. Most studies have explored ways to enhance breeding success, but very few have been carried out during the wintering or postfledging seasons, which are also critical for birds. It is known that ecological circumstances during the nonbreeding seasons (postfledging and wintering) may affect body condition and survival rates (Siriwardena et al. 2000; Stoate et al. 2004), and influence the dynamics of the population (Siriwardena et al. 2007; Butler et al. 2010a,b2010b). Comparisons among seasonal models enabled us to investigate in detail the processes involved in bird responses to AES, that is, which aspects of birds' requirements are better fulfilled by the agri-environmental measures, and which part of their annual cycle is more influenced by these measures. To our knowledge, this is the first study comparing the predictive power of models using field variables of differing quality and exploring the responses of the whole bird community in different seasons.

Our main study hypotheses were that (i) as birds requirements differ throughout different periods of their annual cycle, agri-environmental schemes can lead to different effects in different seasons, and (ii) improving food availability measurements should lead to significantly higher predictive power than just using simple habitat measurements, which sometimes may compensate for the higher field work costs incurred.

## Material and Methods

### Study area, field types, and agri-environmental measures

**Figure 1:**
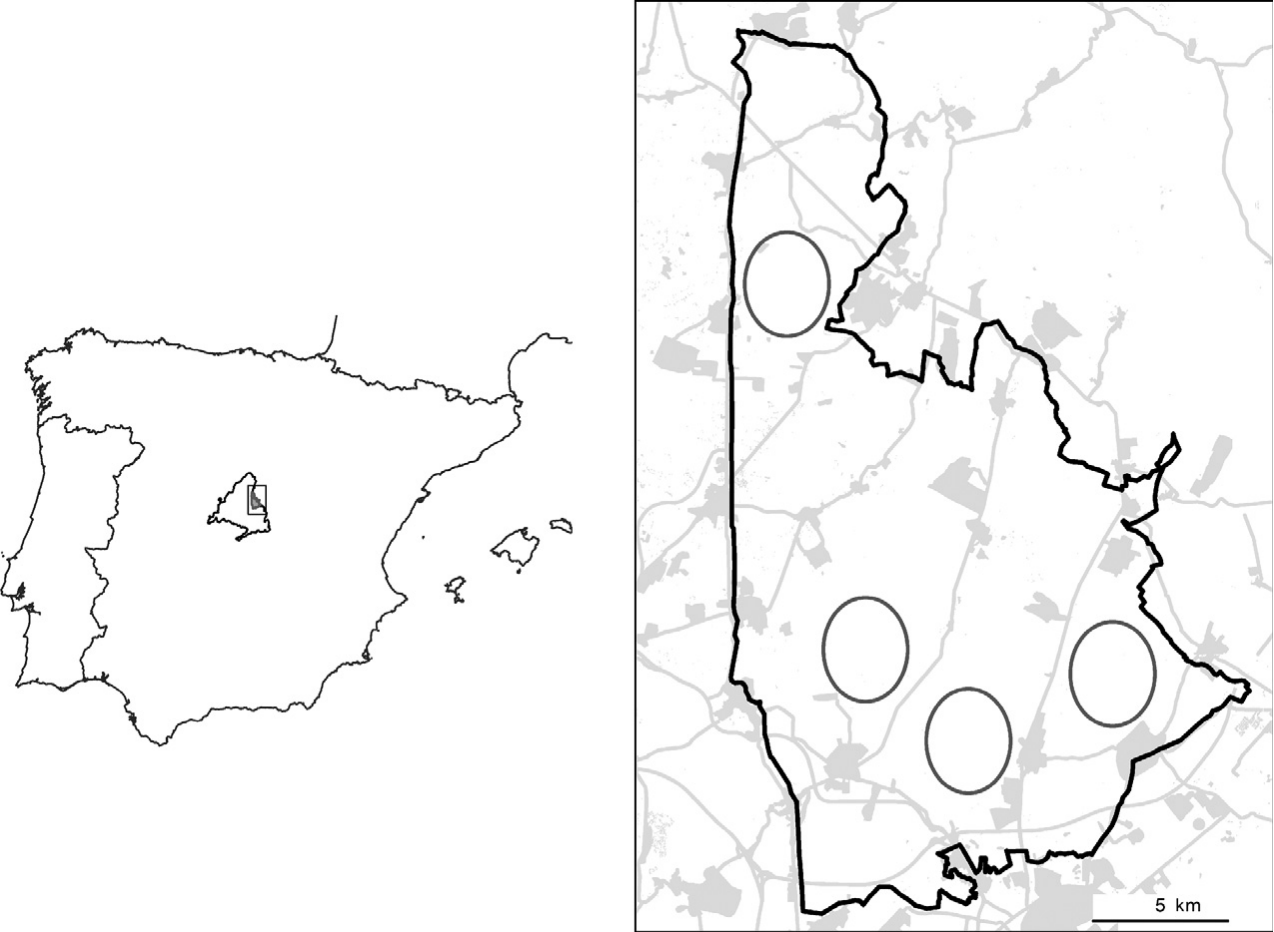
Location of the study area in the Iberian Peninsula. The figure at the right shows the Special Protection Area (SPA) 139 Estepas cerealistas de los ríos Jarama y Henares and the four sites (ellipses) where field work was carried out.

The field work was carried out in the SPA 139 Estepas cerealistas de los ríos Jarama y Henares, located in Madrid region (central Spain), where an AES has been running since 2003. Specifically, we sampled four sites within this area (Fig. 1). The region has dry cereal cultivation as its main land use, and all the sites share major environmental–climatic, biogeographic conditions, as well as a similar steppe bird community.

The study area was a typical nonintensive farmland area, with small fields, margins between neighbor fields and the presence of legumes and cereal stubbles (managed differently from the AES, with the use of pesticides) and fallow fields. Most cereal was grown following a traditional 2-year rotation system (fields are cultivated every second year). We mapped habitat types on GIS-based maps along the transects (200 × 500 m) to calculate the surface of land uses on the same day when bird censuses were carried out. We defined eight field types (Table 1). The agri-environmental measures implemented were maintenance of cereal stubble, growing legumes organically (vetch *Vicia sativa*), and interruption of the cereal production (fallows; Table 1). The managed surface for transects where birds were censused ranged from 0% to 76%. We measured the width of 50 margins at random locations and considered this measure constant throughout the study (Lane et al. 1999).

### Bird surveys

We censused birds from 2006 to 2009 in the winter (between 15 and 31 December), mating (between late April and early May) and postfledging seasons (early July). We carried out one census per season and site (four censuses in each season), which is enough to get reliable information about habitat selection and the above-mentioned parameters (Hanspach et al. 2011). The observer (CP) walked along 9–14 linear transects per site, each 500 m long and 100 m wide at each side of the path (totaling 660 transects (220 in each of the three periods – wintering, mating and post-fledging–) and more than 24,000 birds (see Appendix S1 in Supporting information). Within each site, a distance of 100 m was kept between the end of a transect and the beginning of the next transect. Transects within each site were located at paths that are only used by sheep and farmers. The path within each site was circular and the observer avoided double counting by not considering birds near the beginning and end of this path and looking where flying birds landed during each survey. There was no spatial correlation between consecutive transects in a site (Appendix S2). Birds flying over transects were only taken into account if they were clearly using the field (hunting, hovering, etc.). For each bird flock spotted, we recorded its species composition, number of individuals, and field type where it landed.

### Food availability

Abundance of arthropods, seeds, and vegetation were sampled in three fields of each substrate type, in the four study sites in the SPA from spring 2006 to spring 2008 (three mating seasons, two postfledging seasons and two wintering seasons) to obtain average representative values for each substrate in each season and site. Arthropods were sampled visually following the same methodology as Lane et al. (1999). Two observers walked at low speed (120 m/h, to avoid effects of detectability due to vegetation characteristics) along linear transects (30 m long and 0.5 m wide at both sides) in the middle of each field. We counted and identified every specimen to the most accurate taxonomic level possible by means of visible characters (Oliver and Beattie 1993). We collected a random sample of 7515 arthropods, 12% of the those detected) and assessed the mean biomass of each group using linear regression of weight as function of body length, which was estimated for each individual during the transect (Hódar 1996; Ponce et al. 2011). We measured all collected arthropods in the laboratory. The mean lengths obtained allowed us to estimate the length of the observed and noncollected specimens and to assess the biomass. Finally, for each site, transect, and season, we calculated the surface of each field type and so obtained a weighted average biomass per hectare.

Vegetation abundance and structure were assessed throwing a metal square (25 × 25 cm) at 20 random locations in the middle of each field. Data recorded were total plant cover (%) and height (minimum, maximum, mean and most frequent, in cm). In these samples, we evaluated the roughness of the ground (the degree of flatness of each field) using three categories (low, medium, or high). We also estimated visually the total numbers of seeds on plants and on the ground classifying them in four size categories based on their maximum length (<1 mm, 1–5 mm, 5–10 mm, and >10 mm). We spent the time necessary to count each seed, regardless of the vegetation present or the field type. We collected a sample of seeds to measure, weigh, and estimate the mean biomass for each size in the laboratory. Finally, for each site and season, we calculated the average seed biomass in each substrate type.

The costs for habitat and food models were calculated according to the money and time required to obtain the data. We considered field and laboratory work for food models but only field work for habitat models. Money spent in food models included costs of sampling and weighing seeds and arthropods, measuring vegetation structure and recording field uses. In the case of habitat models, the costs were those of mapping habitat land uses. Both models shared identical bird census costs. We estimated the costs considering salaries, travel, and daily subsistence allowances of observers, and field and laboratory material (excluding those provided by the research center). Time (h) needed for each set of models was calculated according to the effort required for obtaining field and laboratory data.

### Statistical analyses

The response variables were total bird abundance, richness (number of species), Shannon–Wiener diversity index and SPEC-score in each transect, season, and site. The SPEC (Species of European Conservation Concern) is the conservation status of all wild birds in Europe (BirdLife International 2004). The SPEC score index is important because it gives more importance to species of major concern in Europe. Bird species are classified into five categories (from SPEC 1 to SPEC 3 plus Non-SPEC and Non-SPEC^E^ (previously SPEC 4, not detected during the surveys). For the present study, the highest value (4) was assigned to SPEC 1 species (major concern) and the lowest (0) to Non-SPEC species (least concern). The SPEC score was the sum of these values for each different species detected in each transect.

We built averaged mixed models to analyze the response variables. To select the fixed factors in the model, we firstly performed a Principal Component Analysis (PCA), with the Varimax Normalized factor rotation, with all plausible variables in each season (Appendix S3) to explore the degree of association among variables. The percents (%) of field types and vegetation cover were arcsine square root transformed. We only considered axes with eigenvalue >2. We selected the variable that correlated most strongly with the axis for further analyses (always ≥0.7, Appendix S4) to reduce multicollinearity among variables (Barrientos and Arroyo 2014). We preferred to use raw variables instead of PCA factors because their meaning is easier to interpret (Barrientos 2010). This technique allows highly correlated variables to be discarded (which can also be done with simple correlations) and objectively selects the most biologically meaningful and influential variable with each factor (Barrientos 2010). Secondly, we followed the procedure described in the study of Zuur et al. (2009) to select the random factor in mixed models. We built the most complex model (*beyond optimal model*) with all fixed factors from PCA and including different random factors. We considered year, site, both combined, or year nested within site as plausible random factors. To select the random factor, we used the results from the ANOVA test in R-program (R Core Team 2013). We built all possible models for the four response variables in each season using a subsample of the data (154 cases for each season, 70% of the dataset), leaving the rest of data for the validation process. We selected models with an increase in corrected Akaike′s Information Criterion (ΔAICc) over the best model <5 as candidate models (Burnham and Anderson 2002). Finally, with all these models, we performed an average model estimation, with the package MumIN (Barton 2013) in R-program, in which the parameter estimates of all models were combined (Burnham and Anderson 2002). The random factor was that previously selected and the error structure was Poisson for abundance, richness and SPEC-score and Gaussian for diversity. The final averaged models included those variables identified as significant (those whose confident interval excluded 0 value; Alonso et al. 2012; Delgado et al. 2013).

We developed two sets of models for each response variable. The first was that of *food models* which used detailed seed and arthropod biomass measurements and parameters describing the vegetation structure and the surfaces of each field type as candidate variables (see Appendix S3). The second was that of *habitat models*, built using field types and surfaces (Appendix S3). In all cases, the variables selected from each axis had a correlation value ≥0.7 (Appendix S4). We explored the predictive power of each set of models on the 30% previously discarded data set (66 transects distributed evenly among all study sites in each season). This validation shows how accurately the best model predicts data not used before (Seiler 2005; Vaughan and Ormerod 2005). In spite of the acknowledged importance of model validation in behavioral and ecological studies in general, and distribution modeling studies in particular, this issue has been generally ignored in the literature analyzing the efficiency of AES programs.

## Results

### Wintering season

In winter food models, bird abundance was positively predicted by seed biomass found in AES legume fields, surface of AES fallows and ploughed fields, and surface of non-AES cereal stubbles (Table 2). The surface of AES fallows was the most important variable. Bird richness was determined by AES fallow surface, total arthropod biomass, and seed biomass from non-AES high-quality fields (stubbles, fallows, and legume fields; Table 2). Again, the most important variable was the surface of AES fallows. Bird diversity was best explained by a model including the surface of ploughed and cereal stubble fields from regular farming activity (notice: in this case the influence was negative), and the seed biomass from non-AES high-quality fields (non-AES stubbles, fallows, and legumes; Table 2). The SPEC score was determined by seed biomass in legume fields and surface of fallows, in both cases from AES. The standardized regression coefficients showed that AES fallow surface was the most important variable predicting SPEC-score.

**Table 2:** Model-averaged estimates of the food models for bird abundance, richness, diversity and SPEC-score during the wintering, mating and post- fledging periods. The statistics given are: sum of Akaike weights of the models in which the predictor was retained (Σ), parameter estimate of the regression equation (b), standard deviation of the regression parameter (SE), lower and upper confident limits of b, and standardized coefficients of predictors (*β*). Non-significant predictors are not included. Factors are ordered by magnitude of the *β* coefficient

Period	Variable	Parameter	Σ	b	SE	Lower CI	Upper CI	*β*
Wintering	Abundance	Intercept		3.69	0.24	3.22	4.16	0.08
		FallowAES	1	4.36	0.46	3.44	5.28	0.18
		SeedLegAES	1	1.66	0.63	0.40	2.92	0.10
		CerStubbleNAT	1	0.88	0.17	0.54	1.22	0.01
		Plough	0.88	0.43	0.21	0.01	0.85	0.01
	Richness	Intercept		1.27	0.14	0.99	1.55	0.14
		FallowAES	0.87	0.99	0.31	0.37	1.61	0.25
		SeedHQFNAT	1	0.54	0.18	0.18	0.90	0.08
		ArthrTot	0.68	0.19	0.07	0.05	0.33	0.01
	Diversity	Intercept		0.26	0.05	0.15	0.37	0.00
		Plough	1	1.03	0.08	0.87	1.20	0.27
		CerStubbleNAT	1	−0.47	0.10	−0.68	−0.27	−0.16
		SeedHQFNAT	1	0.28	0.09	0.10	0.46	0.08
	SPEC-score	Intercept		2.54	0.16	2.22	2.85	0.10
		FallowAES	1	2.71	0.29	2.13	3.28	0.19
		SeedLegAES	1	1.30	0.32	0.66	1.94	0.10
Mating	Abundance	Intercept		2.22	0.08	2.06	2.38	0.01
		CerStubbleAES	1	2.64	0.49	1.66	3.62	0.11
		LegAES	1	1.84	0.52	0.80	2.88	0.08
		FallowNAT	1	1.36	0.45	0.47	2.25	0.05
		Plough2	1	0.85	0.38	0.09	1.62	0.03
	Richness	Intercept		2.30	0.15	2.01	2.59	0.19
		LegAES	1	1.69	0.12	1.44	1.94	0.12
		FallowNAT	1	1.30	0.16	0.99	1.61	0.11
		LegNAT	0.76	0.69	0.18	0.34	1.05	0.07
	Diversity	Intercept		0.35	0.02	0.30	0.40	0.04
		FallowNAT	1	0.41	0.09	0.22	0.60	0.19
		LegNAT	0.89	0.28	0.09	0.10	0.47	0.12
	SPEC-score	Intercept		2.49	0.20	2.08	2.90	0.08
		FallowNAT	1	0.95	0.40	0.15	1.75	0.06
		Plough2	0.94	0.70	0.34	0.00	1.37	0.04
Post-fledging	Abundance	Intercept		3.20	0.39	2.42	3.98	0.04
		ArthrFallowNAT	0.85	1.93	0.86	0.21	3.65	0.06
		Plough	0.90	−1.12	0.37	−1.86	−0.38	−0.01
		LegStubbleAES	0.75	0.74	0.35	0.04	1.44	0.01
	Richness	Intercept		1.07	0.21	0.65	1.49	0.13
		FallowAES	1	1.26	0.18	0.91	1.62	0.13
		ArthrFallowNAT	0.94	0.67	0.29	0.08	1.26	0.11
		Plough2	1	0.60	0.22	0.16	1.03	0.07
	Diversity	Intercept		0.23	0.04	0.16	0.30	0.03
		FallowAES	0.89	0.30	0.10	0.09	0.50	0.12
		Plough2	0.88	0.31	0.10	0.12	0.50	0.12
		ArthrFallowNAT	0.65	0.24	0.11	0.02	0.46	0.11
	SPEC-score	Intercept		1.38	0.15	1.09	1.68	0.06
		Plough2	1	1.50	0.57	0.36	2.64	0.24
		ArthrFallowNAT	1	0.74	0.32	0.11	1.37	0.07
		FallowAES	0.65	0.55	0.15	0.25	0.85	0.02

AES, agri-environmental schemes; SPEC, Species of European Conservation Concern.

The habitat models for bird abundance and richness included the surface of AES legumes and fallows as predictors, while models for richness and diversity included the surface of non-AES fallows (Table 3). Bird abundance and SPEC score were predicted by the same variables. Also, non-AES cereal stubbles had the lowest effect. The surface of non-AES fallows had a positive effect on bird richness and diversity. Diversity was also positively affected by the surface of ploughed fields (Table 3). Natural or managed fallows had the highest standardized coefficient in all habitat models.

**Table 3:** Model-averaged estimates of the habitat models for bird abundance, richness, diversity and SPEC-score during the wintering, mating and post- fledging periods. The statistics given are: sum of Akaike weights of the models in which the predictor was retained (Σ), parameter estimate of the regression equation (b), standard deviation of the regression parameter (SE), lower and upper confident limits of b, and standardized coefficients of predictors (*β*). Nonsignificant predictors are not included

Period	Variable	Parameter	Σ	b	SE	Lower CI	Upper CI	*β*
Wintering	Abundance	Intercept		4.70	0.13	4.45	4.96	0.05
		FallowAES	1	2.27	0.56	1.14	3.39	0.12
		LegAES	1	1.67	0.25	1.18	2.16	0.04
		CerStubbleNAT	0.87	0.70	0.06	0.58	0.82	3.84E-03
	Richness	Intercept		1.27	0.25	0.77	1.78	0.26
		FallowAES	1	0.68	0.29	0.10	1.27	0.16
		LegAES	1	0.63	0.23	0.17	1.10	0.12
		FallowNAT	1	0.56	0.26	0.04	1.07	0.12
	Diversity	Intercept		0.21	0.08	0.04	0.38	0.06
		FallowNAT	1	0.28	0.12	0.04	0.53	0.11
		Plough	1	0.25	0.11	0.03	0.47	0.09
	SPEC-score	Intercept		2.47	0.17	2.13	2.81	0.10
		LegAES	1	1.00	0.19	0.62	1.37	0.05
		FallowAES	1	0.59	0.26	0.07	1.11	0.04
		CerStubbleNAT	1	0.19	0.08	0.03	0.35	3.78E-03
Mating^1^	SPEC-score	Intercept		1.80	0.18	1.44	2.16	0.05
		LegNAT	1	1.40	0.50	0.40	2.41	0.11
		Plough	1	−0.52	0.14	−0.81	−0.23	−0.01
		FallowNAT	1	0.39	0.19	0.02	0.77	0.01
Post-fledging	Abundance	Intercept		1.80	0.18	1.44	2.16	0.01
		FallowNAT	1	1.73	0.52	0.70	2.77	0.03
	Richness	Intercept		0.88	0.11	0.67	1.09	0.05
		FallowAES	1	0.97	0.27	0.42	1.51	0.15
		FallowNAT	1	0.62	0.29	0.04	1.20	0.10
		Plough2	0.75	0.59	0.25	0.09	1.09	0.08
	Diversity	Intercept		0.22	0.04	0.14	0.29	0.03
		FallowAES	1	0.29	0.10	0.09	0.50	0.12
		Plough2	1	0.24	0.11	0.02	0.46	0.11
		FallowNAT	1	0.20	0.09	0.03	0.37	0.07
	SPEC-score	Intercept		1.37	0.14	1.08	1.66	0.06
		FallowNAT	1	0.82	0.26	0.30	1.34	0.06
		Plough2	1	0.57	0.20	0.17	0.98	0.03

AES, agri-environmental schemes.

1 Food and habitat models retained the same variables.

### Mating season

During the mating season, four significant variables were retained in food models for bird abundance and three for richness, and two diversity and SPEC score (Table 2). The four variables influencing bird abundance had positive effects. Most important were the surfaces of cereal stubbles and legume fields from AES, followed by the surfaces of non-AES fallows and ploughed fields with sprouted weeds.

The best food models for bird richness and diversity included non-AES fallow and non-AES legume surfaces, both showing similar importance (Table 2). The model for bird richness also included AES legume fields as the most influential variable. The averaged model best explaining SPEC score included surfaces of non-AES fallows and ploughed fields with weeds (Table 2). The influence of both variables was positive, fallow surface showing the largest effect.

Habitat models best explaining bird abundance, richness, and diversity during the mating season retained the same predictors as food models (Table 3). The model averaging process showed that three variables influenced SPEC score: surface of non-AES fallows and legumes with positive effects, and surface of ploughed fields, with slightly negative effects.

### Postfledging season

The biomass of arthropods in non-AES fallows was included in final food models for abundance, richness, diversity and SPEC-score (Table 2). The surface of ploughed fields with sprouted weeds was also included in the SPEC-score model with a higher regression coefficient value. Also, the final model for bird abundance included a negative effect of ploughed surface. The final models explaining bird richness and diversity shared all predictors, namely arthropod biomass in non-AES fallows, surface of AES fallows, and surface of ploughed fields with sprouted weeds. In all cases, the amount of AES fallows was the most important variable (Table 2).

Non-AES fallow surface was present in all final habitat models and ploughed land with sprouted weeds was absent only in the model for bird abundance (Table 3). The surface of AES fallows was retained in final models for bird richness and diversity (Table 3). All variables had a positive influence, and those derived from AES showed the highest importance when they were included in the models.

### Comparison of models and their cost

The sensitivity analysis showed that food models had a consistently higher predictive ability than habitat models. The average increase in fit to the data was 13%, reaching a 20% in some variables (Table 4). Fit values were highest for bird abundance and lowest for SPEC-score in both, food and habitat models, and in all three seasons. SPEC score models showed the highest differences in predictive ability between food and habitat models (18% on average). Differences between food and habitat models were usually higher during the postfledging season than during the wintering season.

**Table 4:** Sensitivity analyses for testing the predictive ability of the food and habitat models

		Model predictive ability (%)		
		
Season	Dependent variable	Food	Habitat	Relative increase^1^
Wintering	Abundance	61.8	54.8	12.8
	Richness	56.7	49.3	15.0
	Diversity	50.3	44.9	12.0
	SPEC-score	41.5	34.6	19.9
Mating	Abundance	61.4	–2	–
	Richness	52.7	–2	–
	Diversity	42.9	–2	–
	SPEC-score	39.8	34.6	15.0
Post-fledging	Abundance	51.4	42.4	21.2
	Richness	50.3	41.8	20.3
	Diversity	43.5	36.3	19.8
	SPEC-score	38.4	32.3	18.9

SPEC, Species of European Conservation Concern.

1 Calculated as (food/habitat) × 100.

2 Food and habitat models retained the same variables.

Obtaining food models required almost 2800 h, which was 20 times more than those needed for habitat models (Table 5). Most of this extra time was due to field work (86%). Also, food models needed more than 16,000 € mainly due to the salaries (65%), a cost five times higher than that of habitat models.

**Table 5:** Comparison of costs (in €, work hours or number of people) incurred to measure variables used in food and habitat models

Effort	Food models	Habitat models
People (*n*)	2	1
Field time (h)	2422	135
Laboratory time (h)	372	0
Total time (h)	2794	135
Total time (days)	276	45
Salaries (€)	10,750	2250
Fuel (€)	1830	675
Food (€)	2440	450
Field material^1^ (€)	1286	0
Laboratory material^2^ (€)	105	0
Total cost (€)	16,411	3375

1 Small sampling equipment only.

2 Small laboratory equipment only. A binocular loupe and two optical microscopes were provided by the research institute.

## Discussion

### Food versus habitat models

Habitat and food averaged models retained similar predictor variables, and the main conclusion from both sets of models was that AES benefited steppe birds by increasing the responses analyzed. However, food models were more effective in explaining bird responses, going beyond the simple assessment that AES measures were favorable. First, at least in winter and during the postfledging season, they had a higher predictive power than habitat models (respectively, 15% and 20% higher). Second, they helped inferring important details about the ultimate causes underlying bird responses in different periods of the annual cycle. For example, while during winter habitat models included the surface of fallows, stubbles and legume fields as main predictors, food models revealed the specific importance of seed biomass for most of the response variables. Food models also highlighted the importance of arthropod biomass in fallows during the post-fledging season, when invertebrates are a major component of the diet of juveniles in most bird species. These results show that in two of three seasons birds responded primarily to the amount of food rather than to the surface of fields or the vegetation structure, which was not included in any model. These results support our hypothesis that increasing food resources leads to significantly higher birds numbers for those periods in our study area.

An estimate of the seed biomass, either only in legume fields or altogether in the three substrates considered of high quality (legume fields, stubbles and fallows), was retained in the best winter food models for all response variables investigated. Food models thus captured the important role that seeds play as a source of energy and nitrogen for steppe birds during winter (Evans et al. 2011). The amount of seeds in AES legume fields was particularly important for abundance and SPEC-score models. The positive effect observed on averaged model for the SPEC score means that by increasing the offer of legume fields in winter, the AES program contributed to enhance not only the overall abundance of birds maybe by attracting of local birds to food, but particularly that of species with special conservation interest. This result contradicts the findings of Kleijn et al. (2006), who suggested that endangered species rarely benefited from AES. Richness and diversity of the bird community responded to the total seed biomass in all high-quality substrates (fallows, cereal stubbles, and legume fields) including non-AES fields.

Food models identified the biomass of arthropods as a further variable influencing species richness during winter. That the richness of the steppe bird community was affected by arthropod biomass in winter was a quite unexpected result, as during this season most birds are typically granivorous. This result highlights the importance that arthropods may have even in winter for the bird community of dry cereal farmland in Mediterranean latitudes, whose climatic conditions may favor the presence of arthropod reservoirs available for wintering bird species.

In sum, with the exception of the mating season food models were better than habitat models in predicting bird community responses. In the latter, the direct importance of seeds and arthropods would have gone unnoticed. The gain in predictive power was highest for bird abundance and richness models in summer (respectively, 21.2% and 20.3%), and the SPEC-score model in winter (19.9%). The advantage of a higher predictive power of food models should, however, be balanced against their much higher cost. In this study, the cost of obtaining data for food models was five times higher than for habitat models. Two persons, 130 days field work and 146 days laboratory work (ca. 3000 working hours in total) were necessary to measure the biomass of arthropods and seeds, and the vegetation structure variables. In addition, fuel, materials, and maintenance costs of personnel were also higher in food models. A five times higher cost could appear to be an excessive expenditure, but the additional cost of quantifying food availability may only represent a minor fraction of the total cost of agri-environment programmes.

Our study calls attention to the fact that bird responses could remain unexplained if they are judged only from an assessment of the habitat variables. This could lead to erroneous AES efficiency assessments. In contrast, measurements of food availability and vegetation structure could add important details to help interpreting the reactions of the bird community to AES interventions and thus facilitate evaluating the real efficiency of AES programs. The decision whether to invest in such detailed measurements should be taken considering the specific circumstances of each particular AES program.

### Benefits of AES measures in different seasons

Considering all seasons together, the most effective AES measure was probably the provision and maintenance of fallows. Fallow fields were among the most significant predictors in a majority of food and habitat models in the three seasons. Previous studies already highlighted the importance of fallows in providing food and refuge for several steppe bird species (Duelli and Obrist 2003; Suárez et al. 2004; Billeter et al. 2008). Fallows are perhaps the only substrate offering sufficient amount of varied food types including weeds, seeds, and arthropods (Campbell et al. 1997; Herkert 2009; Lapiedra et al. 2011). It is therefore not surprising that these substrates appear in many AES studies as critical to increase bird abundance, richness and diversity.

Maintenance of cereal stubbles through the winter did not appear to be an AES measure providing a significant benefit to steppe birds, probably because in nonintensive dry cereal areas non-AES cereal stubbles are already abundant in winter. A previous study (Suárez et al. 2004) also suggested that in Spain stubble maintenance through the winter did not benefit farmland birds since these can feed on various non-cultivated substrates. However, in areas where farming is more intensive and thus cereal stubbles scarce or are usually absent in winter, an AES including cereal stubble maintenance may certainly benefit steppe birds (Gillings et al. 2005; Concepción and Díaz 2011; Concepción et al. 2012). In winter food models, the surface of natural cereal stubbles had a positive effect on bird abundance, but a negative effect on bird diversity. This could be so due to the differences in diet among species (Princé et al. 2012), or simply because a marked preponderance of a single species as skylark (*Alauda arvensis*, Appendix S1) implies a reduction of species diversity values. Anyway, raising habitat quality by increasing the amount of food in winter may also produce delayed benefits during the following breeding season (Gillings et al. 2005). Several studies showed that breeding success or fitness of some species were correlated with conditions experienced during the preceding winter (Peach et al. 1999, 2001; Siriwardena et al. 1999, 2000, 2007).

The positive effect of ploughed fields on winter bird abundance and diversity was surprising, given the low values for vegetation cover and arthropod and seed biomass typical of these substrates (this study; see also Díaz and Tellería 1994). However, other authors qualified ploughed fields as important for some bird species (Suárez et al. 2004). For example, wagtails (*Motacilla spp*.) or cattle egrets (*Bubulcus ibis*) follow tractors to feed on invertebrates unearthed during ploughing. A similar behavior has been described for certain granivorous birds that take unearthed seeds (e.g., Whittingham et al. 2006). Finally, certain species may be favored by the lower vegetation cover and the consequent higher antipredator visibility in ploughed fields (Butler et al. 2005), although this possibility has not been tested in this study.

During the mating season, habitat and food models basically coincided. The retained variables were surfaces of various substrates, but no food biomass estimates were included as predictors. This result contrasts with some previous studies (Traba et al. 2008; Concepción and Díaz 2010) which suggest that food availability is a key factor during the mating season. We can think of two possible reasons for the absence of a significant effect of biomass variables during this period of the annual cycle. First, during the mating season, weeds and invertebrates are more abundant in our nonintensive farmland area compared with other periods of the year. Food availability may then be higher than demands and thus represent no limiting factor for bird abundance and diversity. Consequently, the effect of food biomass in each particular substrate type may be obscured during this season. Second, during the mating season, most birds are involved in defending territories, pairing and searching for nest sites. For these tasks, a rough estimate of available surfaces of the different field types may be a better indicator of habitat suitability than a precise estimate of food availability. Substrate selection during this season may indeed provide enough information about the best place to nest and the food availability for chicks expected later in the season. Third, bird abundance, diversity, and richness in spring are limited by other variables such as territoriality, and complex intra- and interspecific interactions within the breeding bird community. In spite of the absence of a clear effect of food biomass during the mating season, the surface of legumes was correlated with the biomass of arthropods in this substrate (0.81), showing its role in increasing food availability. Previous studies had already described the importance of legumes as a source of nitrogen for many species (Karasov 1990), and in particular for steppe birds in dry farmland areas (Bretagnolle et al. 2011; Bravo et al. 2012). Our study suggests that legumes may also be important as a food source for insectivorous species.

Unlike during winter, AES cereal stubbles were the most important predictor in spring averaged model for bird abundance. For birds, natural and AES-managed cereal stubbles are probably identical during most part of the winter, but on managed stubbles, AES restrictions prevent herbicide and insecticide use between harvest and ploughing. This fact surely favored the growth of abundant weeds on managed stubbles in spring and released the observed positive response from birds to the AES stubbles during the mating season. According to the AES rules, ploughing is allowed in January–March, but later forbidden until July. This was an unexpected positive effect of the AES, as we thought all farmers would plough AES stubbles before the deadline of 31 March, as they did with non-AES stubbles (which are also sprayed usually with chemical products against weeds). It is necessary to highlight that AES stubbles were kept from spring to the following autumn without any additional payments to farmers.

During the postfledging season, habitat and food models were also different. While in habitat models, the surface of fallows and ploughed fields with abundant sprouted weeds were the main predictors, food models showed that the birds' response was really induced by the biomass of arthropods in non-AES fallows. In food models, the surface of ploughed fields with sprouted weeds is still retained as a significant predictor. This is because in dry cereal farmland areas ploughed fields with sprouted weeds are the only substrate where birds can find green plants and associated canopy arthropods in summer.

## Conclusions

Our study showed that the AES contributed to increase the abundance and diversity of farmland birds in our study area. The positive responses observed in four variables analyzed were in part induced by some of the AES measures applied. However, many land-use variables not regulated by the AES were also important, probably due to the extensive agricultural regime predominant in our study area. The models presented in our study enabled evaluating the percent benefit obtained from AES measures as compared to non-AES land-use variables.

Exhaustive field work was devoted in this study to measure landscape complexity, vegetation structure and food availability, all of which are considered important factors influencing bird behavior and distribution patterns. In our study, various important effects of seed and arthropod biomass detected using food models would have gone unnoticed using habitat models where only substrate composition is measured. This was at the cost of a much higher investment in time and personnel in food models, with a consequent increase in the total cost of the research. However, detailed food measurements allowed increasing the explanatory power of models describing bird responses, as well as identifying the causes underlying these responses.

Our study finally highlighted the need to apply AES measures and to study bird responses separately in different periods of the year. As most birds use different substrates throughout the annual cycle, the same AES measures may have different effects in different seasons. Thus, farmland areas need to be managed from that seasonal perspective to maximize the benefits of AES programs. In our case, the AES measures aimed at enhancing the benefits of traditional farming cycles at dry cereal areas, by providing supplementary legume crops and fallows and limiting tillage work.

## Supporting Information

**Appendix S1.** List of bird species (in alphabetical order) contacted during surveys of the study area, indicating their total abundance (sum of abundance values in all transects) in each season and the Species of European Conservation Concern (SPEC) category. **Appendix S2**. Correlation values between consecutive transects for each season. **Appendix S3**. Variables defined to measure habitat characteristics and biomass of arthropods and seeds, indicating the model type (food o habitat) in which they were included. **Appendix S4**. Results of the Principal Component Analyses (PCA) carried out to explore correlations among variables.
