# Supplementary material for: Effects of agri-environmental schemes on farmland birds: do food availability measurements improve patterns obtained from simple habitat models?
Source: Ecol Evol. 2014 Jun 11;4(14):2834–47. doi: 10.1002/ece3.1125 (PMC4130443; doi:10.1002/ece3.1125)
Supplement: Supplementary file 1 [file ece30004-2834-SD1.docx]

**Appendix S1**. List of bird species (in alphabetical order) contacted during surveys of the study area, indicating their total abundance (sum of abundance values in all transects) in each season and the Species of European Conservation Concern (SPEC) category.

| Species | SPEC | Wintering | Mating | Post-fledging |
| --- | --- | --- | --- | --- |
| *Accipiter gentilis* | *NON-SPEC* |  |  | 1 |
| *Alauda arvensis* | *SPEC 3* | 8625 | 6 | 5 |
| *Alectoris rufa* | *SPEC 2* | 185 | 108 | 96 |
| *Anas platyrhynchos* | *NON-SPEC* |  | 21 | 1 |
| *Anthus campestris* | *SPEC 3* |  | 14 |  |
| *Anthus pratensis* | *NON-SPEC* | 246 |  |  |
| *Apus apus* | *NON-SPEC* |  | 2 | 9 |
| *Aquila adalberti* | *SPEC 1* |  | 1 |  |
| *Asio flammeus* | *SPEC 3* | 7 |  |  |
| *Athene noctua* | *SPEC 3* |  |  | 3 |
| *Bubo bubo* | *SPEC 3* |  | 1 |  |
| *Bubulcus ibis* | *NON-SPEC* | 29 | 16 | 185 |
| *Burhinus oedicnemus* | *SPEC 3* |  | 66 | 110 |
| *Buteo buteo* | *NON-SPEC* | 10 | 3 | 5 |
| *Calandrella brachydactila* | *SPEC 3* |  | 72 |  |
| *Carduelis cannabina* | *SPEC 2* | 967 | 110 | 77 |
| *Carduelis carduelis* | *NON-SPEC* | 362 | 75 | 121 |
| *Carduelis chloris* | *NON-SPEC* | 99 | 43 | 11 |
| *Ciconia ciconia* | *SPEC 2* |  | 4 | 16 |
| *Circaetus gallicus* | *SPEC 3* |  |  | 5 |
| *Circus aeruginosus* | *NON-SPEC* | 7 | 9 | 6 |
| *Circus cyaneus* | *SPEC 3* | 8 | 6 | 3 |
| *Circus pygargus* | *NON-SPEC* |  | 21 | 19 |
| *Cisticola juncidis* | *NON-SPEC* | 5 | 74 | 52 |
| *Clamator glandarius* | *NON-SPEC* | 3 | 17 | 2 |
| *Columba livia* | *NON-SPEC* | 120 | 98 | 461 |
| *Columba palumbus* | *NON-SPEC* | 33 | 84 | 395 |
| *Corvus corax* | *NON-SPEC* |  | 3 | 1 |
| *Corvus corone* | *NON-SPEC* | 4 |  | 7 |
| *Corvus monedula* | *NON-SPEC* | 29 | 25 | 14 |
| *Coturnix coturnix* | *SPEC 3* | 3 | 102 | 16 |
| *Delichon urbicum* | *SPEC 3* |  | 46 | 45 |
| *Emberiza schoeniclus* | *NON-SPEC* | 4 |  |  |
| *Falco columbarius* | *NON-SPEC* | 7 |  |  |
| *Falco naumanni* | *SPEC 1* |  | 11 |  |
| *Falco peregrinus* | *NON-SPEC* |  |  | 1 |
| *Falco subbuteo* | *NON-SPEC* |  |  | 3 |
| *Falco tinnunculus* | *SPEC 3* | 10 | 9 | 24 |
| *Fringilla coelebs* | *NON-SPEC* | 84 | 6 |  |
| *Galerida cristata* | *SPEC 3* | 139 | 265 | 336 |
| *Gelochelidon nilotica* | *SPEC 3* |  |  | 10 |
| *Hieraaetus pennatus* | *SPEC 3* |  | 1 |  |
| *Hippolais polyglotta* | *NON-SPEC* |  | 1 |  |
| *Hirundo daurica* | *NON-SPEC* |  | 1 |  |
| *Hirundo rustica* | *SPEC 3* |  | 81 | 43 |
| *Lanius meridionalis* | *SPEC 3* | 12 | 4 | 12 |
| *Lanius senator* | *SPEC 2* |  | 3 | 16 |
| *Luscinia megarhynchos* | *NON-SPEC* |  | 1 |  |
| *Melanocorypha calandra* | *SPEC 3* | 524 | 660 | 712 |
| *Merops apiaster* | *SPEC 3* |  | 7 | 16 |
| *Miliaria calandra* | *SPEC 2* | 648 | 564 | 117 |
| *Milvus migrans* | *SPEC 3* |  | 5 | 14 |
| *Milvus milvus* | *SPEC 2* | 4 |  |  |
| *Motacilla alba* | *NON-SPEC* | 98 |  |  |
| *Motacilla cinerea* | *NON-SPEC* |  | 11 |  |
| *Motacilla flava* | *NON-SPEC* |  | 30 |  |
| *Oenanthe hispanica* | *SPEC 2* |  | 78 | 64 |
| *Oenanthe oenanthe* | *SPEC 3* |  | 48 | 31 |
| *Otis tarda* | *SPEC 1* | 541 | 280 | 160 |
| *Parus caeruleus* | *NON-SPEC* | 1 |  |  |
| *Parus major* | *NON-SPEC* |  |  | 1 |
| *Passer domesticus* | *SPEC 3* | 305 | 118 | 165 |
| *Passer hispaniolensis* | *NON-SPEC* | 80 | 52 | 139 |
| *Passer montanus* | *SPEC 3* | 142 | 82 | 61 |
| *Petronia petronia* | *NON-SPEC* | 56 | 42 | 43 |
| *Phoenicurus ochruros* | *NON-SPEC* | 12 |  |  |
| *Phoenicurus phoenicurus* | *SPEC 2* |  | 1 |  |
| *Phylloscopus collybita* | *NON-SPEC* | 4 | 1 |  |
| *Pica pica* | *NON-SPEC* | 169 | 192 | 241 |
| *Picus viridis* | *SPEC 2* | 1 |  | 1 |
| *Pluvialis apricaria* | *NON-SPEC* | 41 |  |  |
| *Pterocles alchata* | *SPEC 3* | 12 | 2 | 9 |
| *Pterocles orientalis* | *SPEC 3* | 84 | 28 | 7 |
| *Saxicola rubetra* | *NON-SPEC* |  | 1 |  |
| *Saxicola torquata* | *NON-SPEC* | 6 | 2 | 14 |
| *Serinus serinus* | *NON-SPEC* | 110 | 98 | 95 |
| *Sturnus unicolor* | *NON-SPEC* | 740 | 186 | 1086 |
| *Sylvia cantillans* | *NON-SPEC* |  | 1 | 3 |
| *Sylvia melanocephala* | *NON-SPEC* | 6 | 3 | 3 |
| *Tetrax tetrax* | *SPEC 1* | 225 | 140 | 63 |
| *Tringa ochropus* | *NON-SPEC* |  |  | 1 |
| *Turdus merula* | *NON-SPEC* | 3 |  |  |
| *Turdus philomelos* | *NON-SPEC* | 31 |  |  |
| *Upupa epops* | *SPEC 3* | 1 | 9 | 16 |
| *Vanellus vanellus* | *SPEC 3* | 368 | 15 | 1 |
| Total birds |  | 15210 | 4066 | 5174 |

**Appendix S2**. Correlation values between consecutive transects for each season. Autocorrelation coefficient values (*ρ*) calculated between each pair of consecutive transects (lag=1) are shown. Asterisks show 0.05>P-values>0.01. There was no autocorrelation coefficient with a P-value <0.01. In each season 64 autocorrelation coefficients were calculated. Because this is a large number of tests, it was expected that a few of them would be significant by chance. Therefore, we applied the Bonferroni correction to the P-values ( Bonferroni-corrected P values were 0.05/64=0.00078) and observed no significant autocorrelation values after correction.

1) Wintering season. Four (6.3%) out of 64 coefficients (marked with *) were significant before Bonferroni correction

|  | Abundance | | | | Richness | | | | Diversity | | | | Spec-score | | | |
| --- | --- | --- | --- | --- | --- | --- | --- | --- | --- | --- | --- | --- | --- | --- | --- | --- |
| Site | 2006 | 2007 | 2008 | 2009 | 2006 | 2007 | 2008 | 2009 | 2006 | 2007 | 2008 | 2009 | 2006 | 2007 | 2008 | 2009 |
| 1 | 0.16 | -0.52 | -0.55 | 0.26 | 0.60* | -0.07 | -0.56 | 0.31 | 0.15 | -0.10 | -0.16 | 0.30 | 0.55 | -0.70* | 0.03 | 0.21 |
| 2 | 0.02 | 0.43 | -0.07 | 0.05 | 0.01 | -0.06 | -0.19 | 0.19 | 0.03 | 0.05 | -0.21 | -0.21 | -0.05 | 0.11 | -0.02 | 0.05 |
| 3 | 0.13 | -0.51 | -0.26 | -0.27 | 0.64* | -0.06 | 0.23 | -0.06 | -0.20 | 0.20 | 0.31 | 0.20 | 0.26 | -0.78* | 0.15 | -0.30 |
| 4 | -0.36 | 0.10 | -0.04 | -0.05 | 0.18 | -0.03 | -0.15 | -0.03 | -0.19 | -0.16 | 0.21 | 0.23 | -0.35 | 0.09 | -0.05 | -0.06 |

2) Mating season. Two (3.1%) out of 64 coefficients (marked with *) were significant before Bonferroni correction

|  | Abundance | | | | Richness | | | | Diversity | | | | SPEC-score | | | |
| --- | --- | --- | --- | --- | --- | --- | --- | --- | --- | --- | --- | --- | --- | --- | --- | --- |
| Site | 2006 | 2007 | 2008 | 2009 | 2006 | 2007 | 2008 | 2009 | 2006 | 2007 | 2008 | 2009 | 2006 | 2007 | 2008 | 2009 |
| 1 | -0.42 | 0.35 | 0.24 | 0.01 | -0.33 | 0.63* | 0.00 | -0.05 | -0.22 | 0.24 | -0.11 | -0.08 | -0.02 | 0.51 | 0.34 | 0.48 |
| 2 | 0.42 | 0.10 | -0.85* | -0.08 | 0.31 | -0.51 | -0.02 | -0.18 | 0.31 | -0.59 | 0.20 | -0.11 | -0.30 | -0.58 | 0.36 | 0.25 |
| 3 | -0.50 | 0.07 | -0.57 | -0.33 | -0.66 | 0.41 | -0.02 | 0.06 | -0.50 | 0.25 | 0.05 | 0.43 | -0.17 | 0.03 | -0.45 | -0.32 |
| 4 | -0.50 | -0.37 | -0.28 | -0.06 | 0.00 | 0.18 | 0.13 | -0.31 | 0.10 | 0.22 | 0.15 | -0.31 | -0.50 | -0.37 | -0.28 | -0.06 |

3) Post-fledging season. One (1.6%) out of 64 coefficients (marked with *) were significant before Bonferroni correction

|  | Abundance | | | | Richness | | | | Diversity | | | | SPEC-score | | | |
| --- | --- | --- | --- | --- | --- | --- | --- | --- | --- | --- | --- | --- | --- | --- | --- | --- |
| Site | 2006 | 2007 | 2008 | 2009 | 2006 | 2007 | 2008 | 2009 | 2006 | 2007 | 2008 | 2009 | 2006 | 2007 | 2008 | 2009 |
| 1 | 0.14 | -0.03 | 0.63* | -0.53 | -0.29 | 0.59 | 0.15 | 0.18 | -0.38 | -0.23 | 0.15 | -0.23 | -0.48 | 0.41 | -0.24 | -0.53 |
| 2 | -0.19 | -0.44 | -0.14 | -0.10 | -0.15 | -0.13 | -0.22 | 0.22 | 0.14 | 0.37 | 0.06 | 0.33 | 0.13 | 0.08 | -0.09 | -0.10 |
| 3 | -0.12 | -0.17 | -0.21 | -0.40 | -0.29 | 0.22 | -0.18 | 0.22 | -0.22 | -0.13 | -0.01 | -0.13 | 0.03 | 0.01 | -0.29 | -0.40 |
| 4 | -0.57 | -0.26 | 0.35 | -0.23 | -0.35 | -0.23 | 0.40 | 0.33 | -0.23 | -0.37 | 0.52 | 0.48 | -0.52 | -0.26 | 0.23 | -0.23 |

**Appendix S3**. Variables defined to measure habitat characteristics and biomass of arthropods and seeds, indicating the model type (food o habitat) in which they were included. Surface variables were measured as percentages per transect, biomass variables as grams per transect, and height as centimetres per transect. All vegetation and ground structure variables calculated for each transect were derived from the mean value in each field type and site and according to its surface.

| Short name | Definition | Model |
| --- | --- | --- |
| ArthrAES | Total arthropod biomass in the sum of all AES fallows,+ AES cereal stubbles + AES legume fields + AES legume stubble fields | Food |
| ArthrCerNAT | Arthropod biomass in non-AES cereal fields | Food |
| ArthrCerStubbleAES | Arthropod biomass in AES cereal stubble fields | Food |
| ArthrCerStubbleNAT | Arthropod biomass in non-AES cereal stubble fields | Food |
| ArthrEdge | Arthropod biomass in edges | Food |
| AES | Surface of AES | Habitat |
| ArthrFallowAES | Arthropod biomass in AES fallow fields | Food |
| ArthrFallowNAT | Arthropod biomass in non-AES fallow fields | Food |
| ArthrHQFNAT | Arthropod biomass in the sum of all non-AES ‘high quality fields’ (non-AES fallows + non-AES cereal stubble + non-AES legumes + non-AES legume stubbles) | Food |
| ArthrLegAES | Arthropod biomass in AES legume fields | Food |
| ArthrLegNAT | Arthropod biomass in non-AES legume fields | Food |
| ArthrLegStubbleAES | Arthropod biomass in AES legume stubble fields | Food |
| ArthrLegStubbleNAT | Arthropod biomass in non-AES legume stubble fields | Food |
| ArthrNAT | Arthropod biomass in non-AES fields | Food |
| ArthrPlough | Arthropod biomass in ploughed fields | Food |
| ArthrPlough2 | Arthropod biomass in ploughed fields with sprouted weeds | Food |
| ArthrTot | Total arthropod biomass | Food |
| CerNAT | Surface of cereal fields | Habitat |
| CerStubbleAES | Surface of cereal stubble fields included in AES | Habitat |
| CerStubbleNAT | Surface of cereal stubble fields not included in AES | Habitat |
| CerStubbleTot | Total surface of cereal stubble | Habitat |
| CoverAES | Mean vegetation cover derived from AES in percentage | Food |
| CoverTOTAL | Mean vegetation cover | Food |
| DifHeight | Difference between the maximum and minimum vegetation height in cm | Food |
| Edge | Surface of edges | Habitat |
| FallowAES | Surface of fallow fields included in AES | Habitat |
| FallowNAT | Surface of fallow fields not included in AES | Habitat |
| FallowTot | Total surface of fallow fields | Habitat |
| HQFNAT | Surface of non-AES ‘high quality fields’ (non-AES fallows + non-AES cereal stubble + non-AES legumes + non-AES legume stubbles) | Habitat |
| LandscapeDiversity | Landscape diversity (Shannon index) | Habitat |
| LandscapeDiversityAES | Landscape diversity (Shannon index) associated with AES | Habitat |
| LandscapeDiversityNAT | Landscape diversity (Shannon index) not associated with AES | Habitat |
| Leg | Total surface of legume fields | Habitat |
| LegAES | Surface of legume fields included in AES | Habitat |
| LegNAT | Surface of legume fields not included in AES | Habitat |
| LegStubbleAES | Surface of legume stubble fields included in AES | Habitat |
| LegStubbleNAT | Surface of legume stubble fields not included in AES | Habitat |
| LegStubbleTot | Total surface of legume stubble fields | Habitat |
| MeanHeight | Mean vegetation height in cm | Food |
| Plough | Surface of ploughed fields | Habitat |
| Plough2 | Surface of ploughed fields with sprouted weeds | Habitat |
| Roughness | Roughness of the ground using three categories (low, medium or high) | Food |
| SeedAES | Seed biomass in AES fallows + AES cereal stubble + AES legume + AES legume stubble | Food |
| SeedCerNAT | Seed biomass in non-AES cereal fields | Food |
| SeedCerStubbleAES | Seed biomass in AES cereal stubble fields | Food |
| SeedCerStubbleNAT | Seed biomass in non-AES cereal stubble fields | Food |
| SeedEdge | Seed biomass in edges | Food |
| SeedFallowAES | Seed biomass in AES fallow fields | Food |
| SeedFallowNAT | Seed biomass in non-AES fallow fields | Food |
| SeedHQFNAT | Seed biomass in non-AES ‘high quality fields’ (non-AES fallows + non-AES cereal stubble + non-AES legumes + non-AES legume stubbles) | Food |
| SeedLegAES | Seed biomass in AES legume fields in AES | Food |
| SeedLegNAT | Seed biomass in non-AES legume fields | Food |
| SeedLegStubbleAES | Seed biomass in AES legume stubble fields | Food |
| SeedLegStubbleNAT | Seed biomass in non-AES legume stubble fields | Food |
| SeedNAT | Seed biomass in non-AES fields | Food |
| SeedPlough | Seed biomass in ploughed fields | Food |
| SeedPlough2 | Seed biomass in ploughed fields with sprouted weeds | Food |
| SeedTot | Total seed biomass | Food |

**Appendix S4**. Results of the Principal Component Analyses (PCA) carried out to explore correlations among variables. The most representative variable from each axis (marked in bold) was selected to be included it in the candidate models for bird abundance, richness, diversity and SPEC-score. The type of model (food, habitat) to which each variable belongs is shown for the first set of models.

1) Food models

| Wintering | Variable |  | PC 1 | PC 2 | PC 3 | PC 4 | PC 5 | PC 6 | PC 7 | PC 8 | PC 9 | PC 10 | PC 11 |
| --- | --- | --- | --- | --- | --- | --- | --- | --- | --- | --- | --- | --- | --- |
|  | ArthrLegStubbleNAT | Food | 0.018 | 0.006 | -0.029 | 0.004 | -0.039 | 0.021 | 0.051 | 0.047 | 0.081 | **-0.968** | -0.003 |
|  | ArthrTot | Food | 0.041 | **-0.954** | 0.014 | -0.066 | 0.011 | -0.063 | -0.022 | 0.003 | -0.069 | 0.015 | 0.001 |
|  | CerStubbleNAT | Habitat | -0.034 | 0.0314 | 0.057 | **-0.933** | 0.005 | 0.042 | -0.049 | -0.121 | 0.068 | 0.010 | 0.061 |
|  | FallowAES | Habitat | **0.871** | 0.021 | -0.061 | 0.029 | -0.08 | -0.016 | 0.142 | -0.066 | 0.167 | -0.132 | 0.049 |
|  | LegNAT | Habitat | -0.063 | -0.031 | -0.019 | 0.042 | 0.023 | 0.019 | 0.030 | -0.051 | 0.024 | -0.121 | **-0.958** |
|  | LegStubbleAES | Habitat | 0.058 | 0.004 | -0.007 | 0.016 | **-0.928** | 0.031 | -0.026 | 0.004 | 0.001 | -0.065 | -0.003 |
|  | Plough | Habitat | -0.091 | -0.002 | -0.132 | 0.231 | 0.046 | 0.122 | 0.158 | 0.066 | **-0.851** | 0.039 | 0.091 |
|  | SeedCer | Food | 0.033 | 0.153 | 0.229 | 0.084 | 0.052 | 0.081 | -0.020 | **-0.768** | 0.084 | 0.02 | 0.144 |
|  | SeedHQFNAT | Food | 0.052 | -0.129 | **0.932** | -0.102 | 0.029 | 0.007 | 0.110 | -0.002 | 0.099 | -0.006 | -0.065 |
|  | SeedLegAES | Food | 0.055 | -0.025 | -0.091 | 0.042 | -0.008 | **-0.937** | 0.056 | -0.031 | 0.054 | 0.032 | 0.066 |

| Mating | Variable | Model | Model | PC 1 | PC 2 | PC 3 | PC 4 | PC 5 | PC 6 | PC 7 | PC 8 |
| --- | --- | --- | --- | --- | --- | --- | --- | --- | --- | --- | --- |
|  | ArthrNAT | Food | Food | 0.086 | -0.174 | 0.014 | 0.486 | -0.021 | 0.084 | **0.835** | -0.061 |
|  | CerStubbleAES | Habitat | Habitat | -0.166 | 0.015 | 0.003 | -0.064 | **-0.951** | -0.066 | -0.036 | 0.021 |
|  | FallowNAT | Habitat | Habitat | 0.033 | -0.249 | 0.012 | **0.931** | 0.085 | -0.055 | -0.022 | -0.034 |
|  | DifHeight | Food | Food | -0.115 | 0.001 | **-0.936** | -0.063 | 0.145 | -0.069 | 0.018 | 0.031 |
|  | LegAES | Habitat | Habitat | **-0.942** | 0.034 | -0.069 | 0.015 | 0.028 | -0.031 | -0.122 | -0.026 |
|  | LegNAT | Habitat | Habitat | 0.068 | 0.081 | -0.049 | 0.138 | 0.103 | 0.081 | -0.016 | **-0.917** |
|  | Plough2 | Habitat | Habitat | 0.165 | -0.121 | 0.006 | -0.068 | 0.057 | **0.908** | -0.006 | 0.009 |
|  | SeedFallowNAT | Food | Food | 0.041 | **-0.947** | -0.011 | 0.280 | 0.021 | 0.038 | 0.016 | 0.012 |

| Post-fledging | Variable | Model | Model | PC 1 | PC 2 | PC 3 | PC 4 | PC 5 | PC 6 | PC 7 | PC 8 | PC 9 |
| --- | --- | --- | --- | --- | --- | --- | --- | --- | --- | --- | --- | --- |
|  | ArthrFallowNAT | Food | Food | -0.043 | **0.921** | -0.049 | 0.018 | 0.071 | 0.01 | 0.003 | 0.001 | -0.006 |
|  | ArthrLegNAT | Food | Food | -0.023 | -0.02 | 0.022 | 0.021 | **-0.995** | 0.004 | -0.009 | -0.03 | 0.024 |
|  | CerStubbleAES | Habitat | Habitat | 0.288 | 0.016 | 0.006 | 0.072 | 0.01 | 0.116 | -0.052 | **0.941** | 0.001 |
|  | FallowAES | Habitat | Habitat | **0.947** | -0.033 | 0.016 | 0.063 | 0.021 | -0.037 | 0.051 | 0.23 | -0.019 |
|  | LegStubbleAES | Habitat | Habitat | 0.261 | -0.016 | -0.008 | -0.103 | 0.01 | **-0.972** | -0.051 | 0.001 | 0.028 |
|  | LegStubbleNAT | Habitat | Habitat | -0.041 | 0.065 | 0.021 | **-0.952** | 0.01 | 0.034 | 0.023 | -0.057 | 0.014 |
|  | Plough | Habitat | Habitat | -0.159 | -0.314 | **-0.912** | 0.098 | 0.004 | 0.006 | 0.152 | -0.089 | 0.086 |
|  | Plough2 | Habitat | Habitat | 0.036 | -0.048 | -0.051 | 0.032 | 0.041 | 0.066 | 0.007 | -0.003 | **-0.943** |
|  | SeedsLegAES | Food | Food | 0.169 | -0.058 | -0.061 | 0.036 | 0.039 | -0.089 | **-0.957** | 0.2162 | -0.001 |

2) Habitat models

| Wintering | Variable | PC 1 | PC 2 | PC 3 | PC 4 | PC 5 | PC 6 | PC 7 | PC 8 | PC 9 | PC 10 | PC 11 |
| --- | --- | --- | --- | --- | --- | --- | --- | --- | --- | --- | --- | --- |
|  | CerStubbleNAT | -0.031 | 0.341 | 0.036 | **-0.933** | 0.007 | 0.041 | -0.051 | -0.112 | 0.059 | 0.002 | 0.061 |
|  | Edge | -0.020 | 0.018 | -0.015 | 0.061 | 0.03 | -0.044 | 0.325 | **0.726** | -0.086 | -0.043 | 0.213 |
|  | FallowAES | **0.872** | 0.014 | -0.046 | 0.039 | -0.052 | -0.015 | 0.21 | -0.074 | 0.201 | -0.141 | 0.061 |
|  | FallowNAT | -0.05 | **-0.723** | 0.312 | 0.165 | -0.031 | -0.052 | 0.468 | 0.219 | 0.044 | 0.036 | -0.019 |
|  | LegAES | 0.351 | 0.01 | 0.102 | 0.002 | 0.051 | **-0.831** | -0.069 | 0.171 | 0.051 | 0.029 | -0.009 |
|  | LegNAT | -0.062 | -0.043 | -0.036 | 0.026 | 0.019 | 0.019 | 0.034 | -0.036 | 0.051 | -0.113 | **-0.972** |
|  | LegStubbleAES | 0.054 | 0.009 | -0.008 | 0.021 | **-0.934** | 0.061 | -0.022 | 0.003 | 0.002 | -0.084 | -0.002 |
|  | LegStubbleNAT | 0.047 | 0.012 | -0.044 | 0.026 | -0.128 | 0.039 | 0.05 | 0.047 | 0.071 | **-0.964** | -0.005 |
|  | Plough | -0.122 | -0.005 | -0.121 | 0.32 | 0.041 | 0.144 | 0.155 | 0.057 | **-0.851** | 0.062 | 0.072 |
|  | SeedFallowNAT | 0.058 | -0.234 | **0.876** | -0.01 | 0.003 | 0.012 | 0.132 | 0.119 | 0.064 | 0.029 | 0.011 |

| Mating | Variable | PC 1 | PC 2 | PC 3 | PC 4 | PC 5 | PC 6 | PC 7 | PC 8 |
| --- | --- | --- | --- | --- | --- | --- | --- | --- | --- |
|  | ArthrCerNAT | 0.21 | 0.121 | -0.232 | -0.253 | -0.068 | 0.018 | **0.731** | -0.056 |
|  | CerStubbleAES | -0.161 | 0.015 | 0.002 | -0.063 | **-0.941** | -0.052 | -0.032 | 0.048 |
|  | FallowNAT | 0.034 | -0.221 | 0.012 | **0.931** | 0.08 | -0.05 | -0.019 | -0.07 |
|  | LegAES | **-0.924** | 0.014 | -0.061 | 0.011 | 0.019 | -0.024 | -0.113 | -0.012 |
|  | LegNAT | 0.071 | 0.049 | -0.041 | 0.131 | 0.051 | 0.082 | -0.032 | **-0.916** |
|  | Plough | 0.037 | 0.18 | **0.861** | -0.166 | 0.135 | -0.158 | -0.12 | 0.063 |
|  | Plough2 | 0.151 | -0.116 | 0.04 | -0.04 | 0.062 | **0.921** | -0.007 | 0.008 |
|  | SeedFallowNAT | 0.026 | **-0.947** | -0.009 | 0.2 | 0.018 | 0.072 | 0.041 | 0.03 |

| Post-fledging | Variable | PC 1 | PC 2 | PC 3 | PC 4 | PC 5 | PC 6 | PC 7 | PC 8 | PC 9 |
| --- | --- | --- | --- | --- | --- | --- | --- | --- | --- | --- |
|  | CerStubbleAES | 0.158 | 0.003 | 0.004 | 0.027 | 0.01 | 0.018 | -0.066 | **0.928** | 0.01 |
|  | FallowAES | **0.961** | -0.017 | 0.012 | 0.051 | 0.008 | -0.035 | 0.079 | 0.26 | -0.039 |
|  | FallowNAT | -0.05 | **0.847** | -0.069 | -0.258 | 0.081 | -0.034 | 0.069 | 0.002 | -0.019 |
|  | LegAES | 0.406 | -0.026 | -0.059 | 0.047 | 0.061 | -0.269 | **-0.915** | 0.121 | 0.011 |
|  | LegNAT | -0.069 | -0.038 | 0.051 | 0.029 | **-0.974** | 0.013 | -0.005 | -0.061 | 0.066 |
|  | LegStubbleAES | 0.174 | -0.041 | -0.012 | -0.019 | 0.006 | **-0.963** | -0.042 | 0.00 | 0.039 |
|  | LegStubbleNAT | -0.072 | 0.109 | 0.009 | **-0.957** | 0.021 | 0.054 | 0.044 | -0.019 | 0.013 |
|  | Plough | -0.151 | -0.301 | **-0.897** | 0.151 | 0.011 | 0.007 | 0.133 | -0.047 | 0.156 |
|  | Plough2 | 0.027 | -0.045 | -0.061 | 0.049 | 0.033 | 0.081 | 0.013 | -0.002 | **-0.927** |
